# Detecting and Classifying Human Touches in a Social Robot Through Acoustic Sensing and Machine Learning

**DOI:** 10.3390/s17051138

**Published:** 2017-05-16

**Authors:** Fernando Alonso-Martín, Juan José Gamboa-Montero, José Carlos Castillo, Álvaro Castro-González, Miguel Ángel Salichs

**Affiliations:** Robotics Laboratory, Universidad Carlos III de Madrid, Av. de la Universidad 30, Leganés, 28911 Madrid, Spain; jgamboa@ing.uc3m.es (J.J.G.-M.); jocastil@ing.uc3m.es (J.C.C.); acgonzal@ing.uc3m.es (Á.C.-G.); salichs@ing.uc3m.es (M.Á.S.)

**Keywords:** acoustic sensing, touch interaction, contact microphone, human-robot interaction, machine learning

## Abstract

An important aspect in Human–Robot Interaction is responding to different kinds of touch stimuli. To date, several technologies have been explored to determine how a touch is perceived by a social robot, usually placing a large number of sensors throughout the robot’s shell. In this work, we introduce a novel approach, where the audio acquired from contact microphones located in the robot’s shell is processed using machine learning techniques to distinguish between different types of touches. The system is able to determine when the robot is touched (touch detection), and to ascertain the kind of touch performed among a set of possibilities: *stroke*, *tap*, *slap*, and *tickle* (touch classification). This proposal is cost-effective since just a few microphones are able to cover the whole robot’s shell since a single microphone is enough to cover each solid part of the robot. Besides, it is easy to install and configure as it just requires a contact surface to attach the microphone to the robot’s shell and plug it into the robot’s computer. Results show the high accuracy scores in touch gesture recognition. The testing phase revealed that Logistic Model Trees achieved the best performance, with an *F*-score of 0.81. The dataset was built with information from 25 participants performing a total of 1981 touch gestures.

## 1. Introduction

The role that the sense of touch plays in emotional communication in humans and animals have been widely studied, finding relations to attachment, bonding, stress and even memory [1,2]. Touch, as a common communicative gesture, is an important aspect of social interaction among humans [3]. Several works focus on using touch as a valid modality to ascertain the user’s intention, and claim about the evidence of the touch as a powerful way of communicating emotions. In this sense, Hertenstein [2] found that anger, fear, disgust, love, gratitude, and sympathy are easier to detect than happiness and sadness.

Hence, touch is revealed as a natural way of interaction that can contribute to improve Human–Robot Interaction (HRI) and promote intelligent behaviour in social robots [4].

Some works have focused on studying the way humans communicate their emotional state to the social robots and the expected reactions when the interaction modality is touch. In this sense, Yohanan et al. [5] presented a touch dictionary of 30 items extracted from social psychology and human-animal interaction literature, identifying which ones are more likely to be used to communicate specific emotions and those which are not. Besides, authors performed a categorization of the human’s higher intents through affective touch, resulting in: protective (*hold*, *hug*, *cradle*); comforting (*stroke*, *rub*, *finger idle*, and *pat*); restful (*massage*, *scratch*, and *tickle*); affectionate (*tickle*, *scratch*, *massage*, *nuzzle*, *kiss*, *rock*, *hug*, and *hold*); and playful (*lift*, *swing*, *toss*, *squeeze*, *stroke*, *rub*, *pat*, *scratch*, *massage*, and *tickle*).

Current social robots commonly include technologies for touch sensing [6]. Some approaches even incorporate touch-gesture recognition although they usually require important hardware deployments mostly directed towards equipping the robot with several sensors. Besides, the reliability of those sensing technologies is limited as most technologies are still dealing with problems such as false positives or low recognition rates. In contrast, our approach is based on analysing the sound vibrations transmitted through the robot’s shell when a touch is performed.

In this paper, we propose exploring contact microphones as sound vibrations propagate much better in solids and liquids than through air. This property is of importance since a contact microphone is able to perceive slight touches in the material, while it is not very affected by ambient noises such as voice. When compared to other approaches in the literature, employing touch microphones seem like a suitable option since this technology is not expensive. More importantly, a single microphone is able to detect events in a solid part of the robot (e.g., head or arm), therefore a reduced number of them would be able to cover a whole robot’s shell. The hardware required is easy to install and configure since it just requires a contact surface to attach the microphone, which is directly plugged into the robot’s computer, to the shell. Moreover, results show high accuracy scores in touch gesture recognition, competitive if compared to the approaches found in the literature.

The rest of the paper is structured as follows: Section 2 reviews the state-of-the-art of touch interaction in Social Robotics. Section 3 describes the proposed system for touch detection and classification. Section 4 details the hardware (robotic platform and microphones) used in this work as well as the set of gestures aimed to be recognized. Section 5 describes the feature collection process with the validation and test of our dataset. Section 6 discusses the results obtained. Finally, the conclusions of this work are presented in Section 7.

## 2. Related Work

The literature on touch detection and recognition offers a wide range of technologies applied to HRI. Additionally, there are proposals focusing on the use of acoustic sensing for touch interaction, although little work directly related to robotics has been done. The following sections delve further into these topics.

### 2.1. Tactile Human–Robot Interaction

The literature offers some interesting reviews presenting proposals for endowing robots with touch detection skills. Among them, a complete survey by Nicholls and Lee [7] can be highlighted.

Even though the contribution is not recent, the authors analysed the state-of-the-art in tactile sensing technology applied to general robots examining in detail the different methods employed for tactile sensing, presenting the pros and cons of these technologies. For instance, sensing technologies such as resistive, capacitive, mechanical, and optical, tend to provide a wide dynamic range with good durability and robustness, although their resolution is in some cases poor (see Appendix A).

A more recent survey was presented by Argall and Billard [6], who surveyed how tactile technologies have been applied to social robots with different materials and shapes. The paper indicated that in the case of robots with hard skins, the most usual sensors are: force/torque, force sensitive resistors (FSR), accelerometers, capacitive sensors, and deformation sensors. Some robots with hard skins include WENDY [8], that incorporated 6-axis force/torque and FSR sensors to detect multiple contacts at the same time, and Maggie [9], which integrated a dozen capacitive sensors that were able to detect just contact. On the other hand, soft-skin robots tend to be equipped with: piezoelectric, potentiometers that provide kinesthetic information, FSR, capacitive sensors, temperature sensors (thermistors), electric field sensors, and photoreflectors. Some robots with soft skins using a fusion of these sensors are Robovie [10], with 276 piezoelectric sensors, Paro [11], which incorporates dielectric and FSR sensors, and CB2 [12], with 197 piezoelectric sensors.

Dahiya et al. presented another interesting review focusing on tactile sensing for human-humanoid interaction [13]. The authors listed and described the main technologies and features applied in this research area, such as: resistive, tunnel effect (these sensors directly convert stress into electroluminescent light and modulates local current density), capacitive, optical, ultrasonics-based, magnetism-based, and piezoelectric.

The works reviewed tend to agree on the fact that the traditional touch sensors used in HRI have some shortcomings, such as requiring almost direct contact or having a short detection range of just a few centimetres from the sensor itself. Therefore, in most approaches it would be necessary to equip the robot with several sensors to cover the whole body of the robot. As an example, the Huggable robot [14] integrates more than 1000 force sensors arranged in a net configuration to detect the amount of pressure exerted, and 400 temperature sensors and nine electric field sensors to determine whether the touch has been performed by a human and to recognize some kinds of touch gestures, such as *tap* to deform the robot surface accordingly.

**Figure 1:**
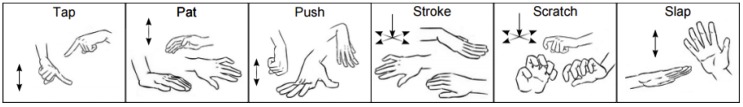
Set of touch gestures using artificial skin defined by Silvera [15].

Recently, Silvera et al. carried out several experiments regarding touch interaction using an artificial arm covered by a sensitive skin layer [15,16]. In this case, the sensing technology is based on electrical impedance tomography, which is sensitive to different types of touch. The acquired information is processed with a LogitBoost algorithm that is able to recognize 6 types of touch (see Figure 1) that are characterized by four features: size of contact area, intensity, in-plane displacement, and duration. Under these conditions, the authors claim to get 74% accuracy over 35 participants, using cross-validation with 1050 instances in the training set.

Following a similar idea, Cooney et al. [17] presented a work that tries to differentiate between 20 affective touches on a humanoid robot. This work is based on artificial vision techniques using Kinect sensors and 14 Kinotex touch sensors (Details about the Kinotex sensor: http://www.esa-tec.eu/workspace/assets/files/12035884441246-51ba009775868.pdf) spread along the robot’s body. Blending these two sensing modes (vision and touch sensors) and using an SVM classifier, the authors claimed to get 90.5% accuracy using cross-validation over its dataset comprising 340 instances by 17 users. Each instance comprised 464 features generated by both the vision and touch modes.

### 2.2. Interaction through Acoustic Sensing

In other areas, touch detection and classification has been successfully addressed using microphones. For example, Paradiso and Checa [18] described a system to locate and classify in a rough way the position of *knocks* and *taps* upon a square glass surface. For that purpose, they used four piezoelectric pickups (also known as contact microphones) placed on the four corners of the material.

They reported high performance, with an accuracy around 2 to 4 cm, also determining the nature of each touch gesture (e.g., *knuckle knock*, *metal tap*, or *bang*). However, that paper does not include details about the underlying technique. Harrison and Hudson [19] introduced a system that allows, through audio analysis, recognizing touches on different objects, especially desks and walls. They claimed that this system can differentiate between six types of touches (*single tap*, *double tap*, *single swipe*, *double swipe*, *tripe swipe*, and *quad swipe*) with an accuracy rate of about 89.5%, validated using a training set composed of 450 touches performed by 15 users. They achieved this using a modified stethoscope which amplifies the captured signal.

Murray-Smith [20] introduced *Stane*, an electronic device controlled by touch interaction based on microphones to operate a music player. For that purpose, *Stane* uses a combination of capacitive and inertial sensors, as well as contact microphones. More specifically, *Stane* can recognize the following touches: stroke, scratch, rub, and tap. The authors argued that the use of piezo-microphones, or contact microphones, is perfectly suitable to collect the vibrations generated for these type of contacts on solid surfaces. For the classification task, they used an artificial neural network trained with 26,880 examples and validated with 11,520 unseen instances (test set). Their experimental results showed an accuracy rate of about 75% using the test set and these four types of contacts. However, that paper did not include information about the number of users participating in the evaluation.

Robinson [21] presented *Tapback*, a system made to interact with call centres on Interactive Vocal Response (IVR) applications, but adding the possibility of tapping the back of the smartphone to enrich the interaction. Traditionally, IVR applications use some technologies to interact with the user: Automatic Speech Recognition (ASR), Text To Speech (TTS), and Touch Tone Dialling (DTMF). Tapback expands these modes adding touch. Tapback recognized whether the user touched the back of his smartphone, in a single tap, twice, or three consecutive times. This interaction allows the user to interact without having to separate the phone from his ear, and the user can continue to listen to speech while performing touches. Specifically, in the application outlined, the touches allowed increasing or decreasing the speed rate of the generated speech. The test set was formed of 1293 instances (the instances were: 772 single, 301 double, and 220 triple *taps*) by 36 users. The authors claimed an accuracy rate of: 1-tap: 93%; 2-tap: 78%; and, 3-tap: 56%. There is no description of the training conditions of the system.

Lopes et al. [22] presented a system that tried to extend the traditional multi-touch systems. The authors mixed two technologies: capacitive sensors to detect the position of the touch, and acoustic sensing to recognize different touch gestures, such as fingers, knuckles, fingernails, and punches. The paper presented no results about the accuracy rate.

Braun et al. [23] proposed a signal analysis and machine learning method that detected four gestures on a surface: swipe, tap, knock, and stomp. They claim an accuracy between 91% and 99% with a single microphone and 97% to 100% with two microphones. The results were obtained using 1247 samples and 10-fold crossvalidation, by 13 users. They considered 587 features of the input audio signal, using the RMS value to detect the beginning and end of a gesture. However, only a SVM classifier was used in classification, without a test set to validate results.

Ono et al. [24] introduced a novel acoustic touch sensing technique called “Touch and Activate”. It required an actuator (the speaker) and an sensor (the contact microphone), both of them attached to the surface of the object. In this way it was possible to recognize some touch contacts with the object like: support, hold it, or grasp. However, this method had some important limitations: it was only applied to small objects and with solid materials like wood, metal, plastic or ceramic.

Harrison et al. [25] presented TapSense. It allows to differentiate interactions with a pen, a finger tip or even a fingernail. They collected data by 18 participants interacting with a multitouch table, making a total of 320 instances. Five kinds of touches were detected: With a pen they classified tip and pad, and with the finger they detected nail and knuckle interaction. The test achieved an accuracy of 88.3% using cross-valitation and the training set.

In this section, we have reviewed several works with the goal of detecting and classifying touch gestures in HRI. It is worth pointing out that those studies usually include few users and a cross-validation over the validation set which is less realistic than using an independent test set.

## 3. System Phases

**Figure 2:**
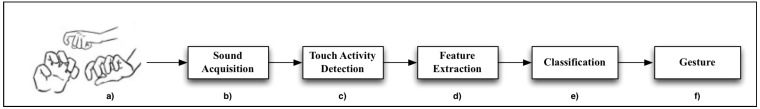
Data flow scheme: (**a**) The touch is produced by the user; (**b**) the vibration is collected by the microphone; (**c**) the beginning of the touch is detected; (**d**) feature extraction phase; (**e**) the ending of the touch is detected; (**f**) the classification phase throws the gesture recognized.

This section describes the implementation of our approach, which is based on three different steps: feature extraction, touch activity detection, and touch classification. Figure 2 offers a summary view of the operation flow. The first step is to determine when the contact begins and ends, in other words, to perform touch activity detection. For that purpose, some thresholds can be fixed using features such as Signal To Noise Ratio (SNR, in dB), Zero Crossing Rate (ZCR), or Volume (computed using the Root Mean Square amplitude, or just RMS, in watts). The window size is of 256 samples, and the sample rate is 44,100 Hz.

These thresholds should be tuned depending on the materials composing the robot’s shell. In our tests, involving acquisition from the solid material of the robot’s shell, the decision rule to differentiate whether the audio samples are noise or a touch is based on the current SNR, $SNR_{c}$ (see Equation (1)), and a dynamic threshold $SNR_{\tau }$ (see Equation (2)). $SNR_{c}$ is computed by dividing the current volume, $RMS_{c}$, by the average noise, $\bar{RMS_{n}}$, as shown in Equation (1). The latter is calculated by averaging the previous $RMS_{c}$ when no touch activity, $T_{a}$, is detected. Besides, $SNR_{\tau }$ is fixed using a step function which we have computed experimentally with respect to the background noise level.

(1) $$\begin{array}{c} SNR_{c}=RMS_{c}/\bar{RMS_{n}} \end{array}$$

(2) $$\begin{array}{c} SNR_{\tau }=\left\{\begin{array}{cc} 75, & if\;\bar{RMS_{n}}<3\\ 25, & if\;(\bar{RMS_{n}}>3\;and\;\bar{RMS_{n}}<10)\\ 10, & if\;(\bar{RMS_{n}}>10) \end{array}\right. \end{array}$$

**Figure 3:**
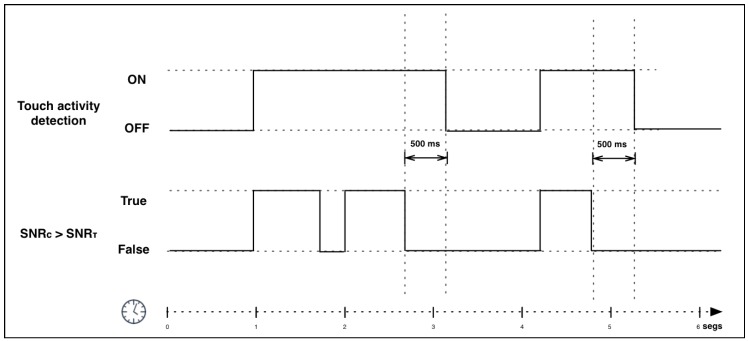
Voice activity detection is based on the relation between the current SNR and a SNR threshold. The beginning of the gesture is detected when the current SNR is greater than SNR threshold and the end of the gesture is detected when SNR is lower than SNR threshold during a fixed period of time.

Using these equations, our system is able to detect the occurrence of a gesture, $T_{a}$, without being affected by external noise. Thus, the system detects touch activity when the current SNR is higher than the threshold, as shown in Equation (3). In order to achieve a more stable output, a 500 ms extra acquisition time begins when $SNR_{c}$ drops below $SNR_{\tau }$ so that touch gestures composed of more than one touch instance (e.g., tickles) are grouped together (see Figure 3). If the extra time expires without detecting touch activity, then the touch is considered finished.

(3) $$\begin{array}{c} T_{a}=\left\{\begin{array}{cc} TRUE, & if\;SNR_{c}>SNR_{\tau }\\ FALSE, & otherwise \end{array}\right. \end{array}$$

**Figure 4:**
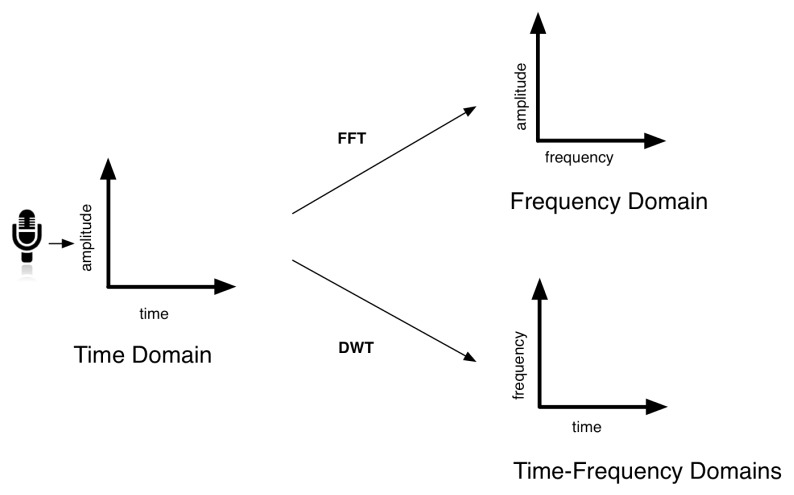
The acoustic signal is analysed in three domains: time, frequency, and time-frequency.

Once the touch activity has been determined, it is necessary to analyse the rest of the features of the sound detected (Take into account that RMS and SNR have already been computed to determine when the contact begins and ends). In this feature extraction process we have used a software component that analyses the acoustic signal in three domains: time, frequency, and time-frequency. This software operates in real time, analysing a series of features extracted from the audio input signal. The ones related to the time domain are directly obtained from the sampled analog signal acquired from the microphone. In the case of features belonging to the frequency domain, the Fast Fourier Transform (FFT) is applied to the time-domain signal [26]. Finally, features related to the time-frequency domain signal are obtained applying the Discrete Haar Wavelet Transform (DWT) [27] (see Figure 4).

The extraction of the signal features has been achieved using an audio processing programming language specifically designed to analyse sound waves, known as Chuck (Chuck website: http://chuck.cs.princeton.edu/). Using Chuck, all the features extracted are computed on-line in a time-window fashion. The features that have been considered are described in Table 1. Once the touch is detected, the feature extraction module calculates the maximum, minimum and mean values of the features described, except for the duration and the number of touches per minute. That makes a total of 23 features per instance. More details about this feature extraction software, known as GEVA, can be found in [28,29].

**Figure 5:**
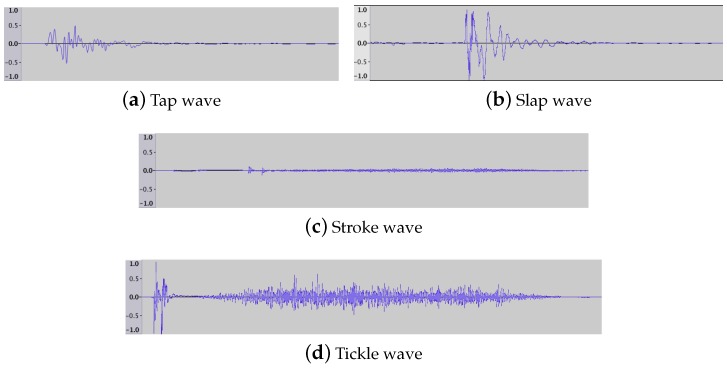
Acoustic signatures for the touch-gestures as acquired by the contact microphone in the time domain. Horizontal axis represents the duration of the sound and the vertical one represents the amplitude normalized by the highest amplitude detected among them.

After the feature extraction, it is necessary to ascertain the kind of contact produced, through a touch classification process. Each kind of touch-gesture generates characteristic sound vibration patterns (*acoustic signatures*) that can be automatically differentiated using machine learning techniques. Figure 5 shows distinctive signatures for the touch-gestures considered in this work regarding duration, intensity level and shape. Using this input information, it is necessary to determine the most accurate algorithm for classifying those touch patterns through their main extracted features. For this task, we have used the software library Weka 3.9 [30] that integrates by default 82 classifiers apart from allowing the incorporation of new ones. In this study we have compared all algorithms included in Weka (A complete list of classifiers available in Weka can be found here: http://weka.sourceforge.net/doc.dev/weka/classifiers/Classifier.html) as well as 44 Weka-based classifiers developed by the community (see the complete list of classifiers added to Weka in Appendix B making a total of 126 classification techniques.

The performance of the classifiers was compared using the *F*-score, which is usually calculating taking into account precision and recall as shown in Equation (4). In our specific case, we used the weighted *F*-score since it takes into account not only the *F*-score of each group to classify (in this case the kind of gesture) but also the number of instances of each group (see Equation (5)). Since both measures are important, it is usual to use the *F*-score as the harmonic mean of recall and precision.

(4) $$\begin{array}{c} F-\mathrm{score}=\frac{2\times Precision\times Recall}{Precision+Recall} \end{array}$$

(5) $$\begin{array}{c} Weighted\;F-\mathrm{score}=\frac{\sum_{i=0}^{number\;of\;classes}\left(F-\mathrm{score}\;of\;i\;\times instances\;of\;i\right)}{total\;instances\;in\;dataset} \end{array}$$

Selecting the best parameters for each algorithm constitutes a complex problem, known as Combined Algorithm Selection and Hyperparameter optimization [31]. The first option would be to manually test all possible classifiers with several configuration parameters. Nevertheless, there is a second approach that allows automating this task using *AutoWeka* [32], a meta-algorithm that automatically finds the *n*-best classifiers with its configuration parameters. Note that currently Autoweka only compares the 82 classifiers integrated by default in Weka. Therefore, we have chosen a mixed approach, using Autoweka for tuning the integrated algorithms, whilst the 44 third-party ones have been manually adjusted.

## 4. System Setup

This section offers some insights into the robotic platform employed in the experiments as well as the sensors used. Finally, the set of gestures recognized by the system is described.

### 4.1. Contact Microphones

In this work we used contact microphones since the sound vibration propagates much better in solids and liquids than through air.

This property is of importance since a contact microphone can perceive slight touches in the material, and is less affected by noises in the environment, such as a voice. For it, we have chosen a contact microphone with high-quality sound acquisition. This model is the *Oyster Schaller 723* (Oyster Schaller microphone website: http://www.schaller-electronic.com/hp541331/Oyster-S-P.htm).

**Figure 6:**
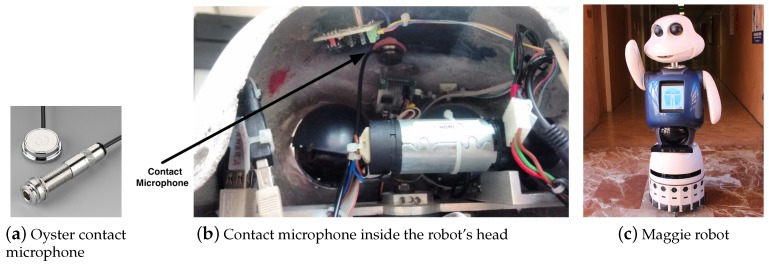
Robotic platform and the integrated contact sensors.

This microphone consists of a polished and chromed oyster-shaped piezoelectric pickup, with a chrome silver cover pre-wired to a standard instrumental cable (see Figure 6a). This device provide advantages, such as that no active circuitry or pre-amplification is required. It also presents a *resistance* of 13.1 KOhm, an *inductance* of 6.4 H, and a *maximum detectable resonance* of 15 dB.

### 4.2. Integration of Contact Microphones in Our Social Robot

The social robot used for this work is the Maggie robot (see Figure 6c). It was developed by the Social Robotics Group at Carlos III University (Madrid) [9,33] as a research platform aimed at research on HRI. Therefore, its external appearance needs to be friendly, with a height of 1.40 m and a wheeled base to move around.

We have placed a contact microphone inside the robot’s head, more specifically, in the inner side of the fibreglass shell (see Figure 6b). Due to the fact that the internal part of the shell is concave and rough, it was necessary to use clay to achieve a smooth and homogeneous surface to maximize the contact between the microphones and the shell. A smooth fitting between the microphone and the shell is crucial to achieve a good sound acquisition.

### 4.3. Set of Touch Gestures

As reviewed in Section 2, there are works in the literature proposing different sets of gestures for HRI. In this regard, Johanan et al. [5] presented a touch set of 30 items extracted from the social psychology and human–animal interaction literature. Similarly, Altun et al. [1] proposed a set of 26 gestures. However, Silvera et al. proposed reducing those sets to 6 gestures [15], discarding some gestures since their aim was to achieve atomic expressions easy to distinguish.

In this work, we have adopted the gesture set proposed by Silvera, considering those more relevant for interaction with a social robot. Thus, push and pat gestures are not considered. On the other hand, stroke is thought to convey empathy, tickle may be associated to fun or joy, tap could transmit warning or advice, and finally slap might be associated to discipline. Table 2 offers a classification of the gestures regarding their contact area, perceived intensity, duration in time and user intention.

Additionally, one of the features that must be highlighted about our approach is the ability for easily incorporating new gestures if new applications require it. This can be achieved by training the classifiers with new sets of touch-gestures.

## 5. Data Analysis

The task of finding the best classifiers could be done in two ways: (i) using *cross-validation* over the training dataset (typically known as validating phase); and (ii) using a different dataset (test set) to validate the system (typically known as testing phase). This test set must be built with new interactions using a different group of users from those who participated in the training dataset. Usually, the second approach gets worse results than the first one, but the accuracy obtained is closer to the one achieved in real interactions. Moreover, the first approach may lead to better results due to a possible overfitting. In this work we follow both approaches, the first one being used to build the classifiers with the training set (Section 5.2), and the second one to validate the accuracy of the classifiers with the test set (Section 5.3). Therefore, we apply the best classifier on the test set in order to evaluate its operation so we can have an idea about our classifier’s performance on non-trained data.

Usually, a training set is about 70–80% and test set about 20–30% of the total amount of samples, according the Pareto Principle (Details about the Pareto Principle: https://www.thebalance.com/pareto-s-principle-the-80-20-rule-2275148), therefore, in this work we have split the data set into 70% and 30%, respectively, for training and testing.

### 5.1. Building the Dataset

The size of a dataset impacts the ability of a system to generalize. In our case, we have collected 1981 touch-gesture instances from 25 different users. This is divided, on the one hand, into a validation set composed by 1347 touch-gesture instances from 10 users. This set is composed by 360 *strokes*, 153 *tickles*, 463 *taps*, and 371 *slaps* and represents 70% of the total amount of instances collected. On the other hand, for the test set we gathered 634 new touch instances performed by 15 users different from the ones used in validation.

In order to homogenize the data acquisition for the dataset, the interactions between the users and the robot Maggie were conducted as follows. The experiment was led by a supervisor. The users involved in the data collection interacted one at a time. The supervisor gave instructions to each user about the areas and the kind of touches to performed. Then, the robot Maggie showed a video tutorial (The video tutorial is available at: http://goo.gl/OUooIG) in its tablet (built-into its chest) showing how to perform one of the gestures mentioned by the supervisor. Then, the users reproduced that gesture on the robot as many times as they wanted. All sounds produced in these interactions, labelled with the kind of gesture performed, were stored to build the dataset. The process was repeated for the four touch-gestures.

At this point, it is necessary to clarify some aspects. First of all, the video tutorial played by the robot before each different kind of gesture was considered as a way to standardize how users should perform the gesture, as people from different cultures could perform gestures in different ways. Second, the user could freely touch the robot at any point inside the area indicated, in this case the robot’s head.

**Figure 7:**
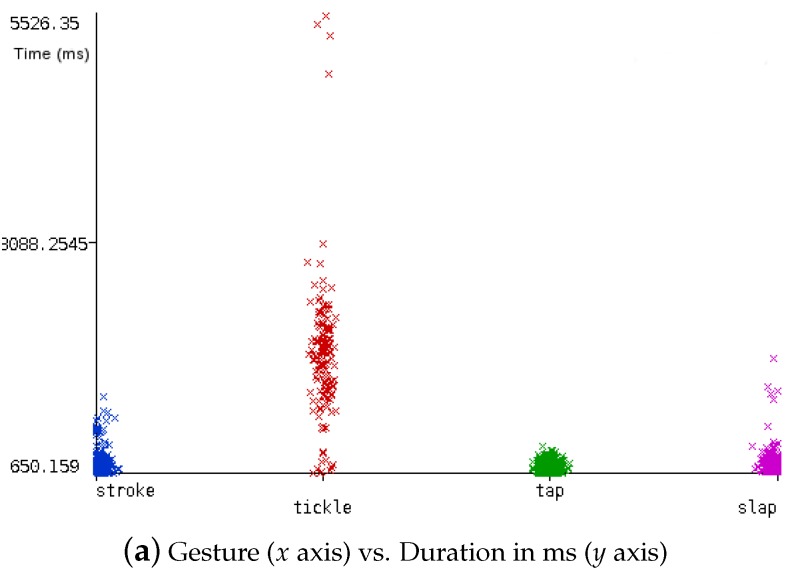

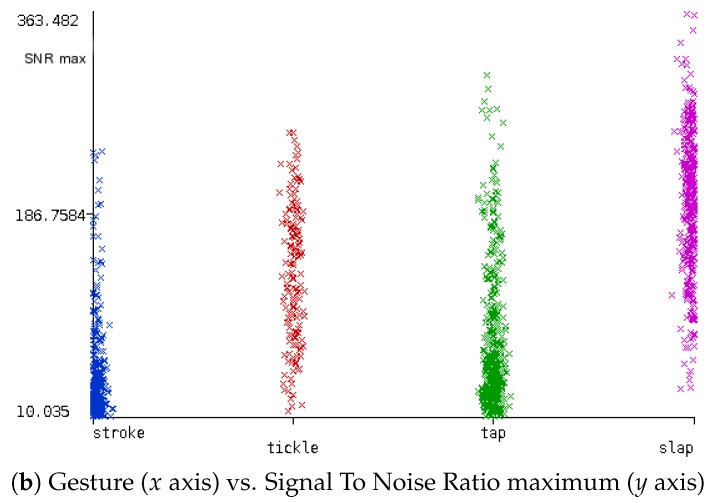
Visual interpretation of the training set.

After gathering all data samples for the dataset, we performed a preliminary phase of analysis to check whether there were differences in the different touch-gestures collected. Figure 7a shows the durations in time of the different gestures. Here we can see how the *tickle* gestures tend to span a longer time period than the others. We also checked the relationship between each kind of gesture and the maximum SNR reached (see Figure 7b). SNR is related to the signal amplitude for each gesture, in other words, how strong a touch-gesture is with respect to the noise.

### 5.2. System Validation

The next step was to find the best classifier to deal with our dataset. To do so, we applied tenfold cross-validation to the training set, a validation technique for assessing how the results of a statistical analysis would generalize to an independent dataset. Usually, five- or tenfold cross-validation are recommended as a good compromise between variance and bias estimating the error [34,35]. These first results (see Table 3) shown no miss classifications for Random Forest (RF) [36], a technique that usually offers good performance.

The second best classifier was the Multilayer Perceptron (MLP) using GreedyStepwise attribute selection, which obtained an *F*-score of 0.93. Logistic Model Trees (LMT) also achieved good performance, although significantly lower than the two previous ones, 0.82 in *F*-score. This approach implements classification trees with logistic regression functions at the leaves.

A similar performance was achieved by a Convolutional Neural Network (CNN) classifier (Johannes Amtén’s CNN implementation: https://github.com/amten/NeuralNetwork). As in the previous case, an SVM-based algorithm provided an *F*-score of 0.80 using Sequential Minimal Optimization [37]. Finally, Deep Learning For Java (DL4J) (DL4S website: https://deeplearning4j.org) was integrated in our Weka framework, showing competitive but lower performance (0.762 *F*-score).

### 5.3. System Testing

From the validation with the initial dataset we obtained some results that seemed too good to be realistic. This was probably caused by overfitting, so in order to check how our trained classifiers are able to generalize, we also tested these classifiers with an untrained part of our dataset, the test set, as described in Section 5.1. Since the users were completely different from the ones forming the first set, the classifiers faced a completely independent set of data.

In this case, the results show how the performance of the LMT classifier almost remained constant regarding the validation stage, with an *F*-score of 0.81 (see Table 4). The performance of RF dropped, giving the second best results, 0.79 in *F*-score, closely followed by a Decision Table/Naive Bayes hybrid (DTNB) approach with a value of 0.78.

## 6. Discussion

Most of the techniques reviewed based their results on applying cross-validation over a validation set usually composed by a reduced number of subjects. This may affect the results in the sense that the trained machine learning techniques are not tested against untrained data. Under similar conditions, using a training dataset composed of 1347 instances collected from 10 users, our proposal with contact microphones and RF obtains a *F*-score of 1. We are aware that this result might result from overfitting, but also 0.93 is achieved for the second best classifier, MLP.

Using a different dataset, composed of 634 instances from 15 users, to test the performance of the system, LMT obtained an *F*-score of 0.81. These results were not as good as those previously obtained using cross-validation, but they are considered more realistic in the sense that the classification instances were completely new to the classifier.

Analysing the results in detail, we found that the top-scored classifiers among the 126 tested coincide with those showing good performance in traditional machine learning works [36,38]. In our case, RF reaches the highest accuracy in validation. However, it is unable to generalize, probably due to overfitting Thus, its accuracy drops when dealing with the test set. In contrast, LMT provides a comparable accuracy both with the validation and the test sets. Apart from that, although the deep learning-based algorithms integrated (CNN and DL4J) performed acceptably, they do not improve on the results achieved by traditional classifiers such as RF or LMT. According to the literature, deep learning algorithms reach their best performance in high-dimensional problems (working with raw data instead of just a set of features) with thousands of samples [39]. Moreover, CNNs are especially suitable for raw image classification [40,41] and raw speech analysis [42].

Going further into the results, Table 5 presents the confusion matrix for the classifier with the highest *F*-score validated against the test set, that is, the LMT classifier. The table shows classification errors obtained in the recognition of each gesture. We can see how, in most cases, the classifier is able to distinguish between the four gestures. However, some of them could be missclassified. For example, stroke gestures are mainly confused with tickles, probably because their duration is alike. Moreover, tap is in some cases confused with strokes because both gestures have low intensity.

These results presented in this paper have some limitations. On the one hand, we built our dataset with a still robot. In this sense, no sound coming from vibrations due to movements interfered with the touch acquisition. In the case of a moving robot, this noise could be filtered by fine-tuning the thresholds described in Section 3. However, raising these thresholds may cause some touch gestures to be undetected, especially faint strokes. Besides, considering that our aim is to use this system in a social robot and most of the interactions are usually performed with a non-moving robot, this limitation does not necessarily constrain the final use of this research. On the other hand, gestures have been collected in a controlled way, since the users were shown a video clip describing how to perform each gesture. We are aware that this data collection scheme might have contributed to raise the system accuracy when compared to unsupervised interaction with the robot, but at this stage we wanted to test the feasibility of the proposal and there is evidence pointing to touch differences regarding culture, gender, age and emotional bonds [43,44]. Finally, the number of users involved (25) and touch instances (1981) may not seem very large. However, this number is comparable to the state of the art as seen in Section 2.

## 7. Conclusions

This paper presented a system, based on machine learning, for detecting and classifying human touch, designed to be applied in a social robot. We proposed using contact microphones due to the fact that they are cheap and a single one is enough to cover an entire part of the robot’s shell (e.g., Maggie’s head). This technology provides rich information when compared to traditional touch-sensing technologies applied to robots, and allows distinguishing between different touch gestures. Apart from that, contact microphones are robust against ambient noise. This aspect is particularly interesting as social robots are intended to coexist with humans, sharing their environment.

Regarding software implementation, the approach is highly modular, allowing the addition of new classifiers or feature extraction techniques in order to achieve better results. Furthermore, the classifiers can be retrained with new information, allowing extending this work to other configurations, including different robots, materials and users.

In this work, we have tested 126 classifiers, tuned to find the best configuration for each one. In order to assess which was the best classifier and how it would perform in a real scenario, the testing was performed in two steps. Firstly, a system validation using cross-validation over a training set demonstrated the feasibility of the proposal, giving high classification rates but with possible overfitting. To address this issue, a second stage was performed using an untrained test set. The accuracy obtained was relatively high, with an *F*-score of 0.81, when compared to the literature.

The methodology and techniques presented in this contribution are intended to endow social robots with the ability to perceive and classify touch gestures, either as a standalone system or complementing traditional touch sensing techniques. 

## Figures and Tables

**Table 1:** The audio features employed in the Feature Extraction component. We are using the maximum, minimum, and average values of pitch, flux, roll-off, centroid, ZCR, RMS, and SNR. Since, a total of 23 features are extracted for each instance/gesture.

Feature	Description	Domain
Pitch	Frequency perceived by human ear.	Time, Frequency, Time-Frequency
Flux	Feature computed as the sum across one analysis window of the squared difference between the magnitude spectra corresponding to successive signal frames. In other words, it refers to the variation of the magnitude of the signal.	Frequency
RollOff-95	Frequency that contains 95% of the signal energy.	Frequency
Centroid	Represents the median of the signal spectrum in the frequency domain. That is, the frequency to which the signal approaches the most. It is frequently used to calculate the tone of a sound or timbre.	Frequency
Zero-crossing rate (ZCR)	Indicates the number of times the signal cross the abscissa.	Time
Root Mean Square (RMS)	Amplitude of the signal volume.	Time
Signal-to-noise ratio (SNR)	Relates the touch signal with the noise signal.	Time
Duration	Duration of the contact in time.	Time
Number of contacts per minute	A touch gesture may consist of several touches.	Time

**Table 2:** Characterization of the touch gestures employed. The last column shows an example of how each gesture can be performed.

Gesture	Contact Area	Intensity	Duration	Intention	Example
**Stroke**	med-large	low	med-long	empathy, compassion	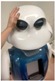
**Tickle**	med	med	med-long	fun, joy	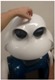
**Tap**	small	low	short	advise, warn	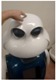
**Slap**	small	high	short	discipline, punishment, sanction	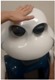

**Table 3:** Classifiers with best performance using the training set and cross-validation.

Classifier	*F*-Score
**RF**	1
**MLP**	0.93
**LMT**	0.82
**CNN**	0.81
**SVM**	0.80
**DL4J**	0.76

**Table 4:** Classifiers with the best performance using the test set.

Classifier	*F*-Score
**LMT**	0.81
**RF**	0.79
**DTNB**	0.78
**MLP**	0.75
**CNN**	0.74
**DL4J**	0.73
**SVM**	0.72

**Table 5:** Logistic Model Trees confusion matrix using the test set composed by 634 new touch instances.

Gesture	Stroke	Tickle	Tap	Slap
**Stroke**	94	21	33	15
**Tickle**	6	122	05	11
**Tap**	8	0	146	7
**Slap**	7	0	04	155

## Appendix A

**Table A1:** Summary of robot sensing technologies.

Sensor Technology	Advantages	Disadvantages
Resistive	-Wide dynamic range. -Durability. -Good overload tolerance.	-Hysteresis in some designs. -Elastomer needs to be optimized for both mechanical and electrical properties. -Limited spatial resolution compared to vision sensors. -Large numbers of wires may have to be brought away from the sensor. -Monotonic response but often not linear.
Piezoelectric	-Wide dynamic range. -Durability. -Good mechanical properties of piezo/pyroelectric materials. -Temperature as well as force sensing capability.	-Difficulty of separating piezoelectric from pyroelectric effects. -Inherently dynamic: output decays to zero for constant load. -Difficulty of scanning elements.-Good solutions are complex.
Capacitive	-Wide dynamic range. -Linear response. -Robust.	-Susceptible to noise. -Some dielectrics are temperature sensitive. -Capacitance decreases with physical size, ultimately limiting spatial resolution.
Magnetic transductor	-Wide dynamic range. -Large displacements possible. -Simple.	-Poor spatial resolution. -Mechanical problems when sensing on slopes.
Mechanical transductor	-Well-known technology. -Good for probe applications.	-Complex for array constructions. -Limited spatial resolution.
Optical transductor	-Very high resolution. -Compatible with vision sensing technology. -No electrical interference problems. -Processing electronics can be remote from sensor. -Low cabling requirements.	-Dependence on elastomer in some designs. -Some hysteresis.

## Appendix B

**Table A2:** Third-party weka classifiers employed.

Name	Developed by	Available on
EBMC	A. Lopez Pineda	https://github.com/arturolp/ebmc-weka
Discriminant Analysis	Eibe Frank	http://weka.sourceforge.net/doc.packages/discriminantAnalysis
Complement Naive Bayes	Ashraf M. Kibriya	http://weka.sourceforge.net/doc.packages/complementNaiveBayes
IBKLG	S. Sreenivasamurthy	https://github.com/sheshas/IBkLG
Alternating Decision Trees	R. Kirkby et al.	http://weka.sourceforge.net/doc.packages/alternatingDecisionTrees
HMM	Marco Gillies	http://www.doc.gold.ac.uk/ mas02mg/software/hmmweka/index.html
Multilayer Perceptrons	Eibe Frank	http://weka.sourceforge.net/doc.packages/multiLayerPerceptrons
CHIRP	Leland Wilkinson	http://www.cs.uic.edu/ tdang/file/CHIRP-KDD.pdf
AnDE	Nayyar Zaidi	http://www.csse.monash.edu.au/~webb/
Ordinal Learning Method	TriDat Tran	http://weka.sourceforge.net/doc.packages/ordinalLearningMethod
Grid Search	B. Pfahringer et al.	http://weka.sourceforge.net/doc.packages/gridSearch
AutoWeka	Lars Kotthoff et al.	https://github.com/automl/autoweka
Ridor	Xin Xu	http://weka.sourceforge.net/doc.packages/ridor
Threshold Selector	Eibe Frank	http://weka.sourceforge.net/doc.packages/thresholdSelector
ExtraTrees	Eibe Frank	http://weka.sourceforge.net/doc.packages/extraTrees
LibLinear	B. Waldvogel	http://liblinear.bwaldvogel.de/
SPegasos	Mark Hall	http://weka.sourceforge.net/doc.packages/SPegasos
Clojure Classifier	Mark Hall	http://weka.sourceforge.net/doc.packages/clojureClassifier
SimpleCART	Haijian Shi	http://weka.sourceforge.net/doc.packages/simpleCART
Conjuntive Rule	Xin XU	http://weka.sourceforge.net/doc.packages/conjunctiveRule
DTNB	Mark Hall et al.	http://weka.sourceforge.net/doc.packages/DTNB
J48 Consolidated	J. M. Perez	http://www.aldapa.eus
Lazy Associative Classifier	Gesse Dafe et al.	http://code.google.com/p/machine-learning-dcc-ufmg/
DeepLearning4J	C. Beckham et al.	http://weka.sourceforge.net/doc.packages/wekaDeeplearning4j
HyperPipes	Len Trigg et al.	http://weka.sourceforge.net/doc.packages/hyperPipes
J48Graft	J. Boughton	http://weka.sourceforge.net/doc.packages/J48graft
Lazy Associative Classifier	Gesse Dafe et al.	http://code.google.com/p/machine-learning-dcc-ufmg/
DeepLearning4J	C. Beckham et al.	http://weka.sourceforge.net/doc.packages/wekaDeeplearning4j
HyperPipes	Len Trigg et al.	http://weka.sourceforge.net/doc.packages/hyperPipes
J48Graft	J. Boughton	http://weka.sourceforge.net/doc.packages/J48graft
Lazy Bayesian Rules Classifier	Zhihai Wang	http://www.csse.monash.edu.au/~webb/
Hidden Naive Bayes classifier	H. Zhang	http://weka.sourceforge.net/doc.packages/hiddenNaiveBayes
Dagging meta-classifier	B. Pfahringer et al.	http://weka.sourceforge.net/doc.packages/dagging
MultilayerPerceptronCS	Ben Fowler	http://weka.sourceforge.net/doc.packages/multilayerPerceptronCS
Winnow and Balanced Winnow Classifier	J. Lindgren	http://weka.sourceforge.net/doc.packages/winnow
Nearest-neighbor-like Classifier	Brent Martin	http://weka.sourceforge.net/doc.packages/NNge
Naive Bayes Tree	Mark Hall	http://weka.sourceforge.net/doc.packages/naiveBayesTree
Kernel Logistic Regression	Eibe Frank	http://weka.sourceforge.net/doc.packages/kernelLogisticRegression
LibSVM	FracPete	https://www.csie.ntu.edu.tw/ cjlin/libsvm/
Fuzzy Unordered Rule Induction	J. C. Hühn	http://weka.sourceforge.net/doc.packages/fuzzyUnorderedRuleInduction
Best First Tree	Haijian Shi	http://weka.sourceforge.net/doc.packages/bestFirstTree
MetaCost meta-classifier	Len Trigg	http://weka.sourceforge.net/doc.packages/metaCost
Voting Feature Intervals Classifier	Mark Hall	http://weka.sourceforge.net/doc.packages/votingFeatureIntervals
Ordinal Stochastic Dominance Learner	Stijn Lievens	http://weka.sourceforge.net/doc.packages/ordinalStochasticDominance
RBFNetwork	Eibe Frank	http://weka.sourceforge.net/doc.packages/RBFNetwork
MODLEM rule algorithm	S. Wojciechowski	https://sourceforge.net/projects/modlem/
The Fuzzy Lattice Reasoning Classifier	I. N. Athanasiadis	http://weka.sourceforge.net/doc.packages/fuzzyLaticeReasoning
Functional Trees	C. Ferreira	http://weka.sourceforge.net/doc.packages/functionalTrees

## References

[1] Altun K., MacLean K.E. (2015). Recognizing affect in human touch of a robot. Pattern Recognit. Lett..

[2] Hertenstein M.J., Verkamp J.M., Kerestes A.M., Holmes R.M. (2006). The communicative functions of touch in humans, nonhuman primates, and rats: A review and synthesis of the empirical research. Genet. Soc. Gen. Psychol. Monogr..

[3] Nie J., Park M., Marin A.L., Sundar S.S. Can you hold my hand? Physical warmth in human-robot interaction. Proceedings of the Seventh Annual ACM/IEEE International Conference on Human-Robot Interaction (HRI).

[4] Ohmura Y., Kuniyoshi Y., Nagakubo A. Conformable and scalable tactile sensor skin for curved surfaces. Proceedings of the 2006 IEEE International Conference on Robotics and Automation (ICRA 2006).

[5] Yohanan S., MacLean K.E. (2012). The role of affective touch in human-robot interaction: Human intent and expectations in touching the haptic creature. Int. J. Soc. Robot..

[6] Argall B.D., Billard A.G. (2010). A survey of tactile human–robot Interactions. Robot. Auton. Syst..

[7] Nicholls H.R., Lee M.H. (1989). A survey of robot tactile sensing technology. Int. J. Robot. Res..

[8] Morita T., Iwata H., Sugano S. Development of human symbiotic robot: Wendy. Proceedings of the 1999 IEEE International Conference on Robotics and Automation.

[9] Gonzalez-Pacheco V., Ramey A., Alonso-Martin F., Castro-Gonzalez A., Salichs M.A. (2011). Maggie: A social robot as a gaming platform. Int. J. Soc. Robot..

[10] Mitsunaga N., Miyashita T., Ishiguro H., Kogure K., Hagita N. Robovie-iv: A communication robot interacting with people daily in an office. Proceedings of the 2006 IEEE/RSJ International Conference on Intelligent Robots and Systems.

[11] Sabanovic S., Bennett C.C., Chang W.-L., Huber L. Paro robot affects diverse interaction modalities in group sensory therapy for older adults with dementia. Proceedings of the 2013 IEEE International Conference on Rehabilitation Robotics (ICORR).

[12] Minato T., Yoshikawa Y., Noda T., Ikemoto S., Ishiguro H., Asada M. Cb2: A child robot with biomimetic body for cognitive developmental robotics. Proceedings of the 2007 7th IEEE-RAS International Conference on Humanoid Robots.

[13] Dahiya R.S., Metta G., Valle M., Sandini G. (2010). Tactile sensing-from humans to humanoids. IEEE Trans. Robot..

[14] Goris K., Saldien J., Vanderborch B., Lefeber D. (2011). Mechanical design of the huggable robot probo. Int. J. Humanoid Robot..

[15] Silvera Tawil D., Rye D., Velonaki M. Touch modality interpretation for an EIT-based sensitive skin. Proceedings of the 2011 IEEE International Conference on Robotics and Automation.

[16] Silvera-Tawil D., Rye D., Velonaki M. (2014). Interpretation of social touch on an artificial arm covered with an EIT-based sensitive skin. Int. J. Soc. Robot..

[17] Cooney M.D., Nishio S., Ishiguro H. Recognizing affection for a touch-based interaction with a humanoid robot. Proceedings of the 2012 IEEE/RSJ International Conference on Intelligent Robots and Systems.

[18] Paradiso J., Checka N. Passive acoustic sensing for tracking knocks atop large interactive displays. Proceedings of the IEEE Sensors.

[19] Harrison C., Hudson S.E. Scratch input: Creating large, inexpensive, unpowered and mobile finger input surfaces. Proceedings of the 21st Annual ACM Symposium on User Interface Software and Technology.

[20] Murray-Smith R., Williamson J., Hughes S., Quaade T. Stane: Synthesized surfaces for tactile input. Proceedings of the Twenty-Sixth Annual CHI Conference on Human Factors in Computing Systems.

[21] Robinson S., Rajput N., Jones M., Jain A., Sahay S., Nanavati A. TapBack: Towards richer mobile interfaces in impoverished contexts. Proceedings of the SIGCHI Conference on Human Factors in Computing Systems.

[22] Lopes P., Jota R., Jorge J.A. Augmenting touch interaction through acoustic sensing. Proceedings of the ACM International Conference on Interactive Tabletops and Surfaces (ITS ’11).

[23] Braun A., Krepp S., Kuijper A. Acoustic tracking of hand activities on surfaces. Proceedings of the 2nd International Workshop on Sensor-Based Activity Recognition and Interaction (WOAR ’15).

[24] Ono M., Shizuki B., Tanaka J. Touch & activate: Adding interactivity to existing objects using active acoustic sensing. Proceedings of the 26th Annual ACM Symposium on User Interface Software and Technology.

[25] Harrison C., Schwarz J., Hudson S.E. TapSense: Enhancing finger interaction on touch surfaces. Proceedings of the 24th Annual ACM Symposium on User Interface Software and Technology (UIST ’11).

[26] Cochran W., Cooley J., Favin D., Helms H., Kaenel R., Lang W., Maling G., Nelson D., Rader C., Welch P. (1967). What is the fast Fourier transform?. Proc. IEEE.

[27] Stanković R.S., Falkowski B.J. (2003). The haar wavelet transform: Its status and achievements. Comput. Electr. Eng..

[28] Alonso-Martin F., Castro-González Á., Gorostiza J., Salichs M.A. (2013). Multidomain voice activity detection during human-robot interaction. International Conference on Social Robotics.

[29] Alonso-Martin F., Malfaz M., Sequeira J., Gorostiza J., Salichs M.A. (2013). A multimodal emotion detection system during human-robot interaction. Sensors.

[30] Holmes G., Donkin A., Witten I. Weka: A machine learning workbench. Proceedings of the Australian New Zealnd Intelligent Information Systems Conference (ANZIIS ’94).

[31] Marques R.Z., Coutinho L.R., Borchartt T.B., Vale S.B., Silva F.J. An experimental evaluation of data mining algorithms using hyperparameter optimization. Proceedings of the 2015 Fourteenth Mexican International Conference on Artificial Intelligence (MICAI).

[32] Thornton C., Hutter F., Hoos H.H., Leyton-Brown K. Auto-WEKA: Combined selection and hyperparameter optimization of classification algorithms. Proceedings of the 19th ACM SIGKDD International Conference on Knowledge Discovery and Data Mining (KDD ’13).

[33] Salichs M., Barber R., Khamis A., Malfaz M., Gorostiza J., Pacheco R., Rivas R., Corrales A., Delgado E., Garcia D. Maggie: A robotic platform for human-robot social interaction. Proceedings of the 2006 IEEE Conference on Robotics, Automation and Mechatronics.

[34] Breiman L. (1992). The little bootstrap and other methods for dimensionality selection in regression: X-fixed prediction error. J. Am. Stat. Assoc..

[35] Hastie T., Tibshirani R., Friedman J. (2009). The Elements of Statistical Learning: Data Mining, Inference, and Prediction.

[36] Caruana R., Karampatziakis N., Yessenalina A. An empirical evaluation of supervised learning in high dimensions. Proceedings of the 25th International Conference on Machine Learning (ICML ’08).

[37] Platt J.C. (1998). Sequential Minimal Optimization: A Fast Algorithm for Training Support Vector Machines.

[38] Fernández-Delgado M., Cernadas E., Barro S., Amorim D. (2014). Do we need hundreds of classifiers to solve real world classification problems?. J. Mach. Learn. Res..

[39] Najafabadi M.M., Villanustre F., Khoshgoftaar T.M., Seliya N., Wald R., Muharemagic E. (2015). Deep learning applications and challenges in big data analytics. J. Big Data.

[40] Krizhevsky A., Sutskever I., Hinton G. Imagenet classification with deep convolutional neural networks. Proceedings of the 25th International Conference on Neural Information Processing Systems.

[41] Lawrence S., Giles C., Tsoi A. (1997). Face recognition: A convolutional neural-network approach. IEEE Trans. Neural Netw..

[42] Palaz D., Magimai-Doss M., Collobert R. (2015). Analysis of CNN-Based Speech Recognition System Using Raw Speech as Input.

[43] Remland M.S., Jones T.S., Brinkman H. (1995). Interpersonal distance, body orientation, and touch: Effects of culture, gender, and age. J. Soc. Psychol..

[44] Suvilehto J.T., Glerean E., Dunbar R.I., Hari R., Nummenmaa L. (2015). Topography of social touching depends on emotional bonds between humans. Proc. Natl. Acad. Sci. USA.

